# Electropolymerization of 5-Indolylboronic Acid: Morphological, Spectroscopic, and Electrochemical Characterization with Perspective Toward Functional Applications

**DOI:** 10.3390/polym17192702

**Published:** 2025-10-08

**Authors:** Danilo Ramos, María Jesús Aguirre, Francisco Armijo

**Affiliations:** 1 Escuela Química, Facultad de Química y de Farmacia, Pontificia Universidad Católica de Chile, Av. Vicuña Mackenna 4860, Macul, Santiago 7820436, Chile; dframos@uc.cl; 2 Millennium Institute on Green Ammonia as Energy Vector—MIGA (ICN2021_023), Pontificia Universidad Católica de Chile, Av. Vicuña Mackenna 4860, Macul, Santiago 7820436, Chile; maria.aguirre@usach.cl; 3 Departamento de Química de los Materiales, Facultad de Química y Biología, Universidad de Santiago de Chile (USACH), Santiago 9170022, Chile

**Keywords:** conducting polymer, electropolymerization, screen-printed carbon electrode, 5-indolylboronic acid, electrochemical sensor, energy storage

## Abstract

Poly(5-indolylboronic acid) was synthesized electrochemically via cyclic voltammetry using various electrodes, including screen-printed carbon electrodes, glassy carbon electrodes, highly oriented pyrolytic graphite, and 304 stainless steel. This study provides a thorough analysis of the resulting conducting polymer’s electrochemical behavior, morphological and structural characteristics, and potential applications. The following techniques were employed: cyclic voltammetry, electrochemical impedance spectroscopy, Fourier-transform infrared spectroscopy, Raman spectroscopy, and field-emission scanning electron microscopy. The polymer exhibits pH-dependent redox activity within the pH range of 4–10, displaying Nernstian behavior and achieving a specific areal capacitance of 0.234 mF∙cm^−2^ on an SPCE electrode. This result highlights the electrode’s efficiency in terms of charge storage. Impedance data indicate that the modified electrodes demonstrate a substantial decrease in charge transfer resistance and improved interfacial conductivity compared to bare electrodes. Contact angle measurements show that the presence of boronic acid groups makes the polymer hydrophilic. However, when 5PIBA was incubated in the presence of molecules containing hydroxyl groups or certain proteins, such as casein, no adsorption was observed. This suggests limited interaction with functional groups such as amino, hydroxide, and carboxyl groups present in these molecules, indicating the potential application of the polymer in biocorrosion. 5PIBA forms homogeneous, stable, and electroactive coatings on various substrates, making it a promising and versatile material for electrochemical technologies, and paving the way for future functionalization strategies.

## 1. Introduction

Conducting polymers (CPs) have been widely investigated for their electrical, optical, and mechanical properties, which position them as versatile materials for electrochemical sensors, electronic devices, energy storage, and functional coatings. Among the synthesis methods, electrochemical polymerization stands out for its cleanliness, control, and ability to directly form films on conductive electrodes by adjusting a fixed potential or current, or number of potentiodynamic cycles, over a given time [1,2,3]. Among the heterocyclic monomers used to obtain CPs are indole derivatives due to their extended conjugation, thermal stability, and ease of functionalization at strategic positions on the benzene or pyrrolic ring, respectively, which allows the structural relationship with the electrochemical properties to be modulated [4,5].

Polyindoles (PIn) have been employed as hybrid materials composed of PIn@reduced graphene oxide (RGO) or PIn@nickel and/or zinc oxides (NiO/ZnO) showing capacitance values of approximately 310 and 323 F∙g^−1^, respectively [6,7]. Also, the capacitive response effect of poly(indole-5-carboxylic acid) (5PICA) [8] and poly(5-cyanoindole) (5PCIn) [9] in different supporting electrolytes has been observed, showing capacitance values of 350 F·g^−1^ and 336 F·g^−1^, respectively. These results indicate the feasibility of applying these materials as supercapacitors.

On the other hand, PIn coatings electrodeposited on 304 stainless steel (SS304) acted as a physical barrier and delayed corrosion processes on the substrate surface, demonstrating excellent protective behavior in different exposure media [10]. In turn, poly(6-aminoindole) obtained on SS304 and used in artificial seawater, has shown significant reductions in Pseudomonas biofilm coverage, proving to be suitable for mitigating marine biocorrosion [11]. PIn have been used as redox active layers in immobilization matrices and porous transducers that favor charge transfer and signal amplification, being actively used in electrochemical sensors and biosensors. An electrochemical immunosensor of poly(indole-6-carboxylic acid) (6PICA) shows a limit of detection (LOD) of 0.8 ng∙mL^−1^ for IgG, demonstrating stability and quantification [12]. Furthermore, functionalizing 6PICA with p-aminoboronic acid allows specific immunosensor platforms with LODs of 0.11 ng∙mL^−1^ and 6 ng∙mL^−1^ to be obtained for the determination of PSA and S20 Proteasome, respectively [13,14]. It was found that boronic groups exhibit a reversible affinity with diols (formation of boronates), which have been widely used for different bioconjugations in different protein immobilizations [15].

Regarding the use of 5-indolylboronic acid, some studies have documented the employment of molecularly imprinted polymer (MIP) films for fluorometric detection of glucose, dopamine, and lactate [16,17,18]. In the realm of chemical oxidation methodologies, particularly those frequently employed in the synthesis of polyindoles, the utilization of FeCl_3_, ammonium persulfate, or analogous oxidants has been identified as a potential source of over-oxidation. This phenomenon, in conjunction with the inability to effectively regulate morphology and thickness, has been shown to result in diminished conductivity and the introduction of reproducibility limitations, which are inherent to the methodology itself. Conversely, an electrochemically produced, self-supporting poly(5-indolylboronic acid) film has been obtained using the monomer in acetonitrile with boron trifluoride diethyl etherate (BFEE) as the supporting electrolyte, yielding a material with flexibility and favorable thermal, mechanical, and electrical properties [19,20,21].

PIn have also been obtained electrochemically in ionic liquids due to their wide working potential window and the possibility of incorporating bulky anions as dopants, with improvements in film density and conductivity [22]. In acidic aqueous media (H_2_SO_4_/HClO_4_), the doping process and C2–C3 coupling are favored during electropolymerization [20], with the possibility of in situ growth monitoring. However, overoxidation has been documented at high potentials or prolonged times, requiring careful control of experimental conditions [5,8,20,21,22,23,24]. Studies using LiClO_4_ as a support electrolyte in acetonitrile have achieved adherent, homogeneous films with reproducible p-type doping, making this system a baseline option for exploring the polymerization of new monomers [25].

However, indole monomers with a boronic substituent group at different positions of the benzene ring have received scant attention, and their applications have been limited. A systematic study of the electrochemical oxidation of these monomers is required to establish the optimal conditions for the production of indole-boronic polymers. The utilization of these PIns holds considerable promise for applications in energy storage, sensors, and coatings.

This work addresses the electrochemical synthesis of poly-5-indoleboronic acid (5PIBA) by cyclic voltammetry on different substrates, along with its structural, morphological, and electrochemical characterization. The motivation is twofold. On the one hand, the boronic group provides Lewis acidity and reversible chemistry with diols, enabling intrinsic selectivity (e.g., toward saccharides and catechols) and dynamic crosslinking useful in sensors and adaptive materials; in addition, it facilitates the anchoring of ions/oxides to generate electroactive hybrids and, in coatings, provides adhesion and additional chemical functionality. Electrochemical production is especially advantageous. It allows uniform films to be deposited on the electrodic surface; the thickness, morphology, adhesion, and compatibility with substrates of interest to be controlled; and a clean and scalable route to be opened to integrate boron polyindoles into sensors, corrosion/biocorrosion, and advanced electrochemical devices.

## 2. Materials and Methods

### 2.1. Materials and Reagents

All reagents were of analytical grade and used without further purification. The monomer 5-indolylboronic acid, lithium perchlorate (LiClO_4_), 2-morpholinoethanesulfonic acid sodium salt (MES), D-(+)-glucose, potassium hexacyanoferrate(II), potassium hexacyanoferrate(III), and other chemicals were purchased from Sigma-Aldrich (St. Louis, MO, USA). Acetonitrile (ACN, HPLC grade) was obtained from PanReac (Barcelona, Spain) and Applichem (Darmstadt, Germany). Blocker Casein in PBS (blocking buffer) was purchased from Thermo Fisher Scientific (Waltham, MA, USA). Recombinant human MIF protein was obtained from the Human MIF DuoSet ELISA kit (Cat. #DY289, R&D Systems, Bio-Techne, Minneapolis, MN, USA). All solutions were prepared using Milli-Q grade water (18 MΩ·cm^−1^). Screen-printed carbon electrodes (SPCE, DRP-110) and their connectors (DRP-CAC and DRP-DSC) were obtained from Metrohm DropSens (Oviedo, Spain).

### 2.2. Equipment

The equipment used for the experiments included a BioLogic VSP-300 potentiostat/galvanostat. The morphological characterization of the resulting coatings was determined by FESEM (Quanta Feg250 scanning electron microscope in a high vacuum mode under an acceleration voltage of 5 kV, Thermo Fisher Scientific, Waltham, MA, USA). Fourier-transform infrared spectroscopy (FT-IR) and attenuated total reflectance (ATR) measurements were carried out using Thermo Fisher Scientific Nicolet is-10 equipment (Thermo Fisher Scientific, Waltham, MA, USA). Subsequently, the samples were examined using a WITec Alpha 300 RA Raman spectrometer. Contact angle measurements were performed using a Contact Angle Goniometer L2004A1, Ossila, Sheffield, UK.

### 2.3. Electrochemical Synthesis

The electropolymerization of 5-indolylboronic acid was carried out using a 10 mM solution of monomer and 0.1 M of LiClO_4_ in acetonitrile (ACN). Screen-printed carbon electrodes (SPCE), glassy carbon electrodes (GCE), highly oriented pyrolytic graphite (HOPG), and 304 stainless steel (SS304) were used as working electrodes (1.0 cm^2^ was the surface in contact with the solution in each experiment). A platinum wire was used as the auxiliary electrode, while a Ag/AgCl (KClsat) electrode was used as the reference electrode. Electrochemical experiments were carried out using three-compartment electrochemical cells, applying 20 potential cycles between –0.4 V and +1.2 V at a scan rate of 80 mV·s^−1^. For the SS304 electrode, a potential window of –0.6 V to +1.4 V was employed.

### 2.4. Electrochemical Characterization

Electrochemical characterization was performed by cyclic voltammetry (CV) and electrochemical impedance spectroscopy (EIS). EIS measurements were performed over a frequency range of 10 kHz to 10 mHz, with an amplitude of 10 mV and 10 points per decade, at open circuit potential (OCP) and at the formal potential of the [Fe(CN)_6_]^3−^/^4−^ redox couple. CV experiments were conducted within a potential window of −0.5 V to +0.5 V at a scan rate of 100 mV·s^−1^. All electrochemical studies were carried out in MES (pH 5) or PBS (pH 7), purged and maintained under a nitrogen atmosphere.

### 2.5. Electrochemical pH Response

The influence of pH on the electrochemical response was studied on SPCEs within a potential window of −0.6 V to +0.3 V at a scan rate of 100 mV·s^−1^. Britton–Robinson buffer solutions in the pH range of 4 to 10 were employed.

### 2.6. Interactions with Biomolecules

An exploratory evaluation of the interactions between 5PIBA and biomolecules was performed by incubating of 1.0 mg·mL^−1^ glucose, μg·mL^−1^ MIF and, 1% P/V casein for 1 h. CV was carried out in the presence of 5 mM [Fe(CN)_6_]^3−^/^4−^ at a scan rate of 100 mV·s^−1^.

## 3. Results and Discussion

### 3.1. Electrochemical Synthesis

**Figure 1:**
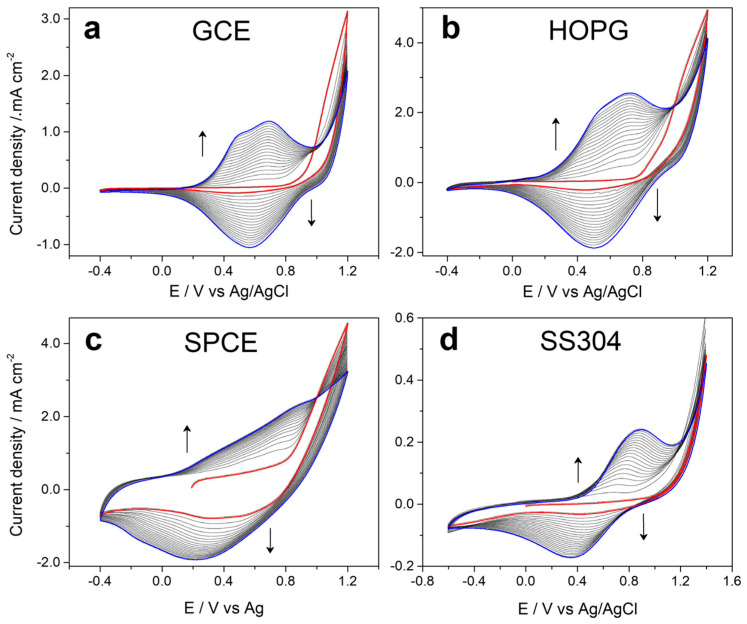
Cyclic voltammograms recorded during 20 cycles of 5-indoleboronic acid electropolymerization in the potential range from −0.4 to 1.2 V at 0.08 V·s^−1^ from a 1 × 10^−1^ mol·L^−1^ EDOT + 1 × 10^−1^ mol·L^−1^ LiClO_4_ solution in CH_3_CN on (**a**) GCE, (**b**) HOPG, (**c**) SPCE, and (**d**) SS304.

Figure 1 shows the voltammetric profiles obtained during the electrochemical production of 5PIBA on different substrates—(a) GCE, (b) HOPG, (c) SPCE, and (d) stainless steel—using a 10 mM monomer solution in acetonitrile with 0.1 M LiClO_4_ as the supporting electrolyte. It is observed that on GCE, HOPG, and SPCE in the first cycle (red line), the monomer presents an oxidation initiation potential at approximately 0.8 V vs. Ag/AgCl and then in consecutive potentiodynamic cycles in the range of −0.4 V to +1.2 V at a scan rate of 80 mV∙s^−1^, with a progressive growth of the anodic and cathodic charge until cycle 20 (blue line), with the formation of an electroactive polymeric film on the surface of each electrode. Faradaic processes are of the doping/undoping type, in which the polymer is oxidized by incorporating anions from the electrolyte, and when it is reduced, it expels them in the cathodic potential sweep. The symmetry between each cycle suggests efficient polymerization on these carbon surfaces, favored by their π-conjugated nature and good surface conductivity [12,13,14,26,27]. This behavior has been observed for indoles dissolved in ACN and LiClO_4_ as supporting electrolytes [12,13,14].

On the other hand, in the case of the stainless steel electrode (Figure 1d), it was necessary to extend the anodic potential sweep range to +1.4 V to induce effective monomer oxidation and allow the growth of the 5PIBA polymer. This requirement is attributed to the passivating nature of steel, which spontaneously forms a surface film of metal oxides, which acts as a dielectric barrier and prevents direct electron transfer from the metal surface to the monomer, thus demonstrating the low total charge obtained compared to other substrates. Furthermore, metallic electrodes such as steel typically have a higher oxidation overpotential compared to carbon electrodes due to their lower density of electronic states available for charge transfer, which requires greater energy to generate the cationic radicals responsible for the initiation of polymerization. Added to this is the lower surface affinity of steel for aromatic organic compounds, which limits the initial adsorption of the monomer and delays polymer nucleation [28,29,30]. Despite the differences in the electrochemical nature of the electrode surfaces used, the results demonstrate that 5PIBA can be efficiently obtained by forming polymeric films. This is based on the monomer’s versatility and suitability as a precursor to a conducting polymer, reaffirming its potential as a functional material in the development of electrochemical platforms for advanced applications.

### 3.2. FESEM Morphological Characterization

**Figure 2:**
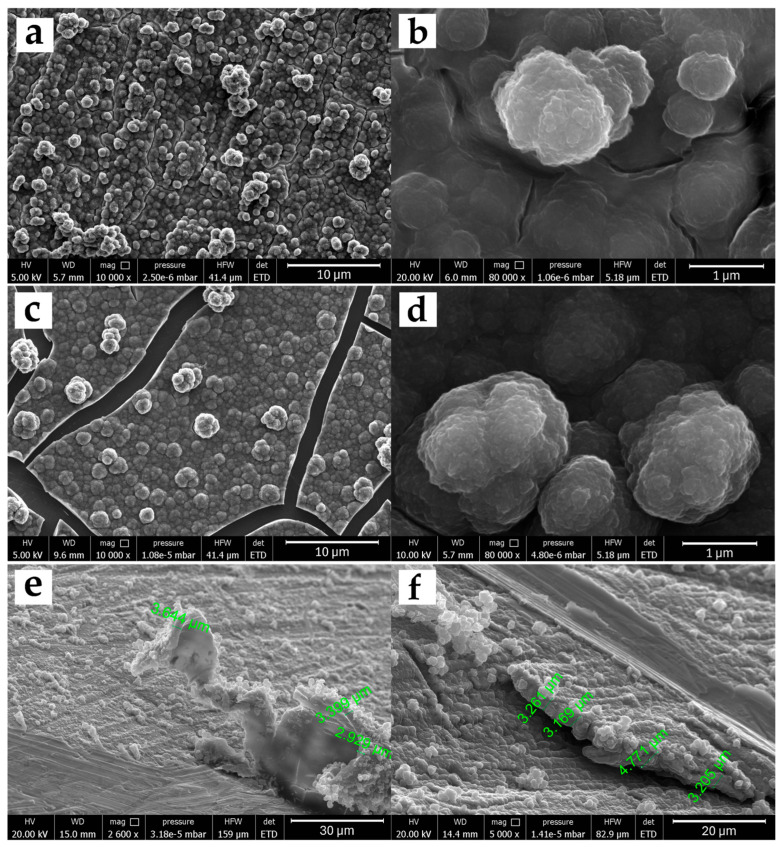
FESEM micrographs of 5PIBA films on SS304 (**a**,**b**) and HOPG (**c**,**d**), acquired at 10,000× (**left panels**) and 80,000× (**right panels**). Panels (**e**,**f**) show exploratory scratch cross-sections on SS304 (30 polymerization cycles); the irregular delamination and substrate damage indicate that this destructive approach is not suitable for reliable film-thickness quantification (scale bars as shown).

Figure 2 shows the images obtained by FESEM microscopy for the morphological study of the 5PIBA polymer on stainless steel (Figure 2a) and HOPG (Figure 2c). Before performing the FESEM measurements, the modified surfaces were subjected to a drying stage, where they were subjected to temperature under vacuum conditions and accelerated dehydration, producing fracture zones only in the polymer film obtained on HOPG due to contraction processes or mechanical stress. On both surfaces, a three-dimensional granular morphology is observed with granular domains of different sizes, a tendency toward coalescence, and a higher density of superimposed aggregates. The aggregates are well-defined, spherical, and densely distributed over the surface (Figure 2b,d). This type of structure is characteristic of many conducting polymers synthesized by electrochemical methods and has been reported for systems such as polypyrrole or polyaniline, where granular aggregation promotes an increase in surface area and high porosity [31,32]. A study of the nucleation and growth mechanism of polypyrrole under the same experimental conditions on steel found that the formation of these granular structures is influenced by two contributions, an instantaneous nucleation with two-dimensional growth (IN2D) and a progressive three-dimensional nucleation with diffusion-controlled growth (PN3Ddif), which favor the formation of compact layers during polymer growth [33,34,35].

The effect of the substituent on the benzene ring on the morphology was observed when poly-5-indolecarboxylic acid (5PICA), poly-6-indolecarboxylic acid (6PICA), and poly-7-indolecarboxylic acid (7PICA) were obtained under equivalent electrochemical conditions [12,13,14,36]. The formation of randomly intertwined fiber-like structures of different sizes was found, very different from the granular structure observed for poly-6-aminoindole [11], similar to that presented by 5PIBA in this study. This difference is due to the nature and position of the functional group on the indole; while the carboxyl group favors the formation of nanowires, the presence of an amino group or a boronic group in the 5-position induces more ordered layer nucleation and favors the formation of spherical aggregates. This observation suggests that small structural changes in the monomer can have a significant impact on the final polymer architecture, even when the polymerization conditions are similar.

From a functional perspective, these morphologies present desirable characteristics for electrochemical applications. The three-dimensional granular structure provides a high electroactive surface area, which favors ionic transport and the access of redox species to the interior of the film, and improves doping and dedoping processes. Furthermore, the greater homogeneity and organization of the film can translate into improved mechanical properties and greater reproducibility of the coating, which is relevant for the development of stable and functional electrochemical sensors or platforms based on 5PIBA. To complement the plan-view analysis, a scratch cross-section thickness estimate was performed (Figure 2e,f). Cross-sections were produced on SS304 (30 polymerization cycles) by mechanically scribing the coating, and the exposed edge was imaged by FESEM. Along the scribed boundary, the 5PIBA layer appears to be continuous, with local thicknesses ranging from ~2.93 to 4.77 µm, as measured at multiple locations. These values are reported as first-order, location-specific estimates, given that the scribing procedure can induce partial delamination and substrate damage that blur the true interface. Consequently, these values should not be taken as an absolute thickness of the coating.

### 3.3. FTIR and RAMAN Spectroscopic Characterization

**Figure 3:**
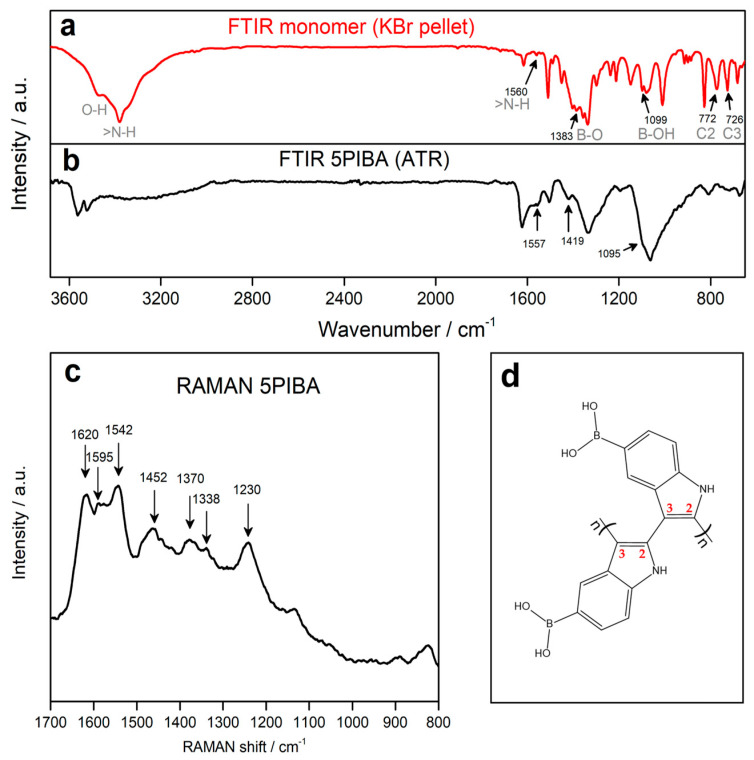
(**a**,**b**): FT-IR spectra of the monomer *5-indoleboronic acid* and their corresponding polymer 5PIBA. (**c**) RAMAN spectrum of 5PIBA. (**d**) Structure proposed C2–C3 coupling during 5PIBA formation.

As illustrated in Figure 3a,b, the FTIR spectra of the monomer (5-indolylboronic acid) and 5PIBA are presented. For the monomer, a broad band is observed between the 3200 and 3600 cm^−1^ region, presenting two absorption peaks at 3490 and 3400 cm^−1^ corresponding to the O–H and N–H stretching vibrations in the molecular structure. The two peaks exhibit a shift and overlap, with a position of 3570 cm^−1^ and 3520 cm^−1^, respectively, as observed in the 5PIBA spectrum. The absorption peak observed at 1557 cm^−1^ is attributed to the in-plane bending of the N-H bond, indicating the presence of N-H groups within the polymer. The absorption peaks at 1419 and 1095 cm^−1^ for 5PIBA and at 1383 and 1099 cm^−1^ for the monomer correspond to the asymmetric vibration of the B-O bond and the bending mode of the B-OH group, respectively [16,19]. The absorption peaks at 772 and 726 cm^−1^ in the monomer are attributed to the out-of-plane deformation vibrations of the C2-H and C3-H bonds of the pyrrole ring [19]. These peaks almost disappear in the 5PIBA spectrum, suggesting that the polymerization sites occur at the C2 and C3 positions on the pyrrole ring (see Figure 3d). This finding is consistent with spectroscopic data reported by nuclear magnetic resonance (NMR) and Fourier-transform infrared spectroscopy (FTIR), indicating the same polymerization sites of other 5-substituted polyindoles [5,19,20,21].

After polymerization, the FTIR spectrum of 5PIBA continues to display the bands observed in the monomer spectrum, with small shifts and broadenings, which can be attributed to the formation of a conjugated polymer network and the change in the electronic environment of the material. In particular, the persistence of the broad band in the 3400–3600 cm^−1^ region confirms the presence of boronic groups (-B(OH)_2_) in the polymer structure, suggesting that these substituent groups on the benzene ring remain after the polymerization process. However, the relative decrease in this band may be associated with the confinement of the boronic groups in the polymer matrix [19,36,37,38].

Figure 3c shows the Raman spectrum of 5PIBA, allowing the identification of the main signals associated with the indole ring and the π-conjugated system of the polymer. The spectrum shows bands at 1230, 1338, 1370, 1452, 1542, and 1595 cm^−1^, assigned to stretching vibrations of C=C and C=N bonds in the aromatic skeleton. Additionally, a well-defined band is observed at 1620 cm^−1^, characteristic of the C–N stretching of the indole ring [39,40,41,42,43]. In Raman spectroscopy, the vibrations associated with the boronic group (-B(OH)_2_) appear below 800 cm^−1^ [44]. However, the conservation of the main bands of the indole ring suggests that the polymerization does not involve the boronic substituent groups, which is in agreement with the results obtained by FTIR. This route has been widely described for substituted indoles, where the electronic orientation and steric factors determine the final polymer topology [42,45,46]. The 2,3 coupling results in an angular arrangement between monomers, which favors branching and the formation of partially ordered three-dimensional networks, producing well-defined spherical aggregates.

### 3.4. Surface Wettability Characterization

**Figure 4:**
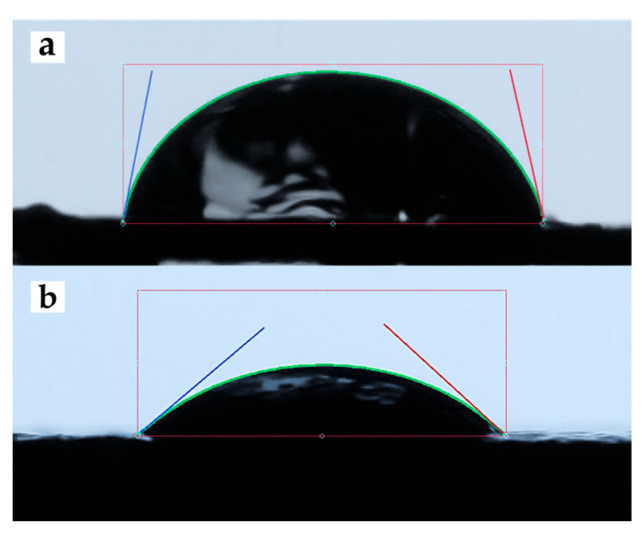
Contact-angle images of (**a**) clean HOPG and (**b**) 5PIBA-coated HOPG.

Figure 4 shows a representative image of the contact angle measurement of 5PIBA deposited on HOPG. Table 1 summarizes the contact angle values obtained for unmodified HOPG and HOPG modified with 5PIBA. The bare HOPG exhibited an average contact angle of 79.11° (Figure 4a), corresponding to moderately hydrophilic behavior, typical of surfaces with low surface energy and limited interaction with water [45].

Subsequent to the formation of a 5PIBA film on HOPG, a decrease in the water contact angle was observed, reaching 38.17° (Figure 4b). This finding indicates a discernible shift toward hydrophilic behavior (θ < 90°). Across a total of n = 4 electrodes that were prepared independently, the mean contact angle was found to be 38.17 ± 4.03° (SD), with a coefficient of variation (CV) of approximately 10%, suggesting that the repeatability is deemed to be acceptable. In comparison with clean HOPG (79.11°; single measurement), this corresponds to an approximate 52% reduction, which is consistent with the introduction of polar functionalities in the coating. These polar functionalities include boronic acid groups [-B(OH)_2_] and indole N–H, which increase surface free energy and wettability. This behavior is relevant from a physicochemical perspective; greater wettability favors the diffusion and penetration of electrolytes into the polymer matrix, which is a key factor in the efficiency of doping/undoping processes and charge transfer. Therefore, the combination of a porous morphology with high roughness and a highly hydrophilic surface suggests that the 5PIBA polymer has optimal characteristics for electrochemical applications.

### 3.5. Electrochemical Characterization of 5PIBA Film

**Figure 5:**
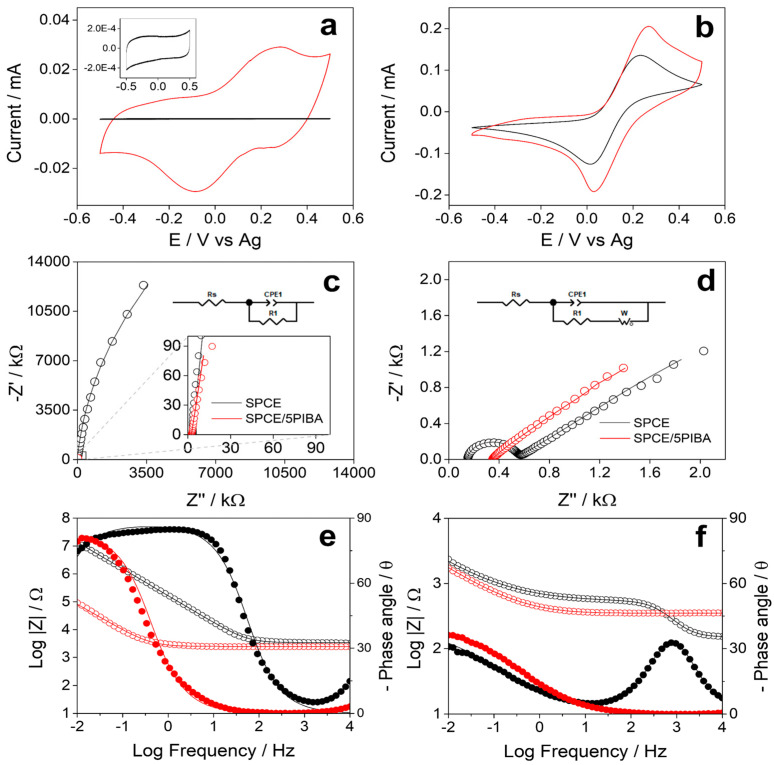
Electrochemical characterization of bare SPCE (black) and SPCE/5-PIBA (red) in MES buffer (pH 5.0) for CV and EIS (**a**,**c**,**e**) without redox probe and (**b**,**d**,**f**) with 5 mM K_3_[Fe(CN)_6_]/K_4_[Fe(CN)_6_].

Figure 5a shows the stable electrochemical response obtained for the unmodified SPCE (black line) and for the 5PIBA-modified SPCE (red line) in MES buffer solution (pH 5). The bare SPCE shows only a capacitive response in the investigated potential range, without associated redox processes (inset Figure 5a). In contrast, the 5PIBA/SPCE exhibits a well-defined redox process in the potential range of −0.5 to 0.5 V. The scan rate study was performed in MES buffer between 0.02 and 0.2 V∙s^−1^. It was observed that the anodic and cathodic peak currents increased with increasing potential scan rate, suggesting a process controlled by adsorption of the polymer onto the surface (data not shown). This behavior has also been reported for other conducting polymers [42,45,46]. To quantify the charge-storage capacity, the total charge associated with the SPCE/5PIBA redox response was determined (Q = 0.029 mC). Using a geometric area of 0.1256 cm^2^, an areal capacitance of 0.234 mF·cm^−2^ was calculated. This value exceeds that of purely capacitive materials such as activated carbon (0.009–0.011 mF·cm^−2^) and lies within the range expected for pseudocapacitive polymers [47,48]. It should be emphasized that this capacitance corresponds strictly to films grown with five polymerization cycles and characterized at 100 mV·s^−1^ in MES buffer within a conservative potential window; because capacitance depends critically on the electrolyte, potential window, scan rate, film loading (mass loading) and porosity, direct comparison with supercapacitor oriented reports (high-ionic-strength/acidic media, wider windows, larger loadings or nanostructured supports) is not strictly equivalent. Even under these mild, aqueous conditions, the value is promising and suggests that targeted optimization of film loading and electrolyte could translate into superior performance. Table 2 compares these data with other polymers reported in the literature, supporting the idea that the formed layer is an attractive coating for charge-storage applications or integration into supercapacitors.

Additionally, a VC and EIS study was carried out under the same experimental conditions in MES buffer at pH 5 and in the presence of 5 mM [Fe(CN)_6_]^3−^/^4−^ to evaluate the redox response of 5PIBA. Figure 5b shows that SPCE and SPCE/5PIBA electrodes exhibit a well-defined redox signal with an anodic to cathodic current ratio (Ia/Ic) close to 1, indicating good process reversibility. In the presence of the redox couple, 5PIBA exhibits an increase in peak currents compared to those obtained for bare SPCE, indicating improved charge transfer of the modified surface. Figure 5c shows the Nyquist plots at open circuit potential (OCP) for SPCE (black line) and for SPCE/5PIBA (red line) in MES buffer solution (pH 5); the data fitting is shown in Table 3. In the frequency range from 10 mHz to 10 KHz, both show a capacitive behavior, adjusting to a Randels equivalent circuit (insert Figure 5c) that includes the dissolution resistance (Rs), the charge transfer resistance (R1), and a constant phase element (CPE1) that represents the capacitance of the double layer due to non-faradaic resistive processes by the accumulation of ions on interfacial heterogeneities of the electrode material, CPE1 presents an increase of two orders of magnitude, from 10^−6^ F to 10^−4^ F, for SPCE/5PIBA and a decrease of one order of magnitude for R1 compared to bare SPCE, corroborating that 5PIBA can be used as a charge storage material. Furthermore, the impedance spectra (Figure 5d) show that the low-frequency response is controlled by diffusion of the dissolved species toward the electrode surface. To fit these data, it was necessary to add a Warburg impedance to the equivalent circuit (inset Figure 5d). This impedance is particularly important in systems where the diffusion of ionic species to the electrode/solution interface plays a crucial role, such as in batteries and electrochemical cells.

The Bode plots were obtained in the absence (Figure 5e) and presence (Figure 5f) of 5 mM Fe(CN)6]^3−^/^4−^. In the MES buffer (pH 5), the SPCE/5PIBA (red) demonstrates a lower Z modulus value in comparison to the unmodified SPCE (black) within the intermediate to low frequency range (Figure 5e). Conversely, in the presence of the redox couple, the Z modulus value attains a comparable level for both electrodes across the frequency range examined (Figure 5f). In both cases, the Z modulus for SPCE/5PIBA demonstrates consistent behavior at high and intermediate frequencies. This indicates that there is an absence of a predominant capacitive or inductive effect that modifies the impedance with frequency. A decreasing Z modulus value indicates a low impedance, suggesting that the circuit offers minimal resistance to the flow of electric current. The characteristic diffusion regime at low frequencies with a phase angle close to 45° is presented for both electrodes in the presence of the redox couple (Figure 5f). In general, the Bode plots appear to support the proposed equivalent circuits, thereby demonstrating that the 5PIBA film improves interfacial capacitance and facilitates charge transfer. In these systems, the supporting electrolyte and the electropolymerization protocol have been shown to govern the nucleation and growth mechanisms, doping/dedoping, and the film morphology [5,25,36].

Table 3 shows the best parameters obtained by fitting the impedance data, presenting a χ^2^ < 0.001. In the presence of the redox couple CPE1, the CPE1 increase by three orders of magnitude, from 10^−6^ F to 10^−3^ F, for SPCE/5PIBA, and there is a decrease of several orders of magnitude for R1 compared to bare SPCE. Consequently, 5PIBA is positioned as a highly electroactive functional coating for applications in electrochemical sensors and biosensors, given its high conductive capacity, generating a stable and reproducible electrochemical response.

Polypyrrole is one of the polymers that exhibit granular morphologies when obtained by electrochemical methods [30,31,32,33,34], with homogeneous, adhesive, and electroactive characteristics comparable to those of polyindoles. However, a significant benefit of VC is its capacity to enable potentiodynamic charge control on a cycle-by-cycle basis by modulating the potential sweep rate. The process under investigation has been demonstrated to promote the formation of ordered and uniform structures on an electrode surface with well-defined Faradaic characteristics. It also enables the regulation of the applied potential window, thereby averting overoxidation, a process that can result in non-conductive materials [12,22,23,24].

### 3.6. Influence of pH on Electrochemical Behavior

**Figure 6:**
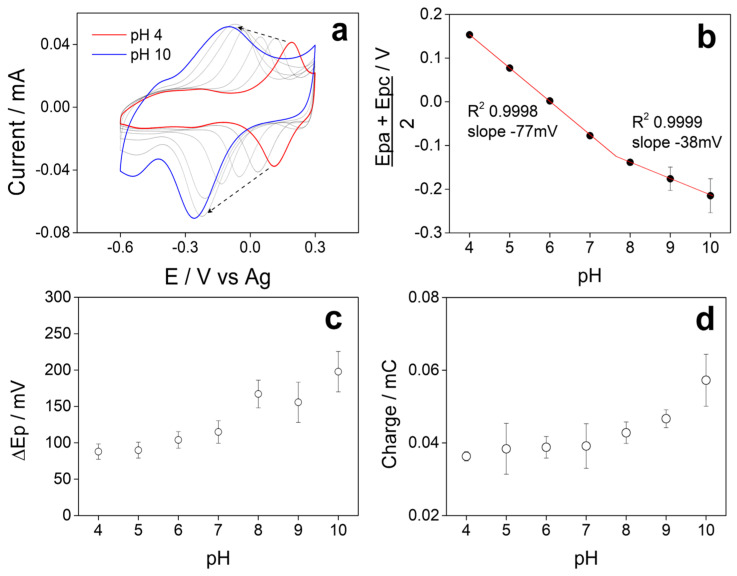
(**a**) Voltammetric profiles at different pH of 5PIBA in Britton–Robinson buffer. Plots of the variation (**b**) (Epc + Epa)/2, (**c**) ∆Ep, and (**d**) total charge as a function of pH.

Figure 6a shows the stable voltammetric profiles obtained in Britton–Robinson buffer at different pH of SPCE/5PIBA in a potential window between −0.6 V and 0.3 V at a scan rate of 100 mV s^−1^, where we observe in each VC, a single redox couple that shifts towards more negative potentials when the pH of the solution increases; this is characteristic behavior of a coupled proton–electron (H^+^/e^−^) charge transfer process [54,55,56]. Figure 6b shows the pH vs. (Ep + Epa)/2 graph; two linear regions are observed between pH 4–7 with a value of −77 mV/pH, adjusting to a Nernstian behavior that involves a H^+^/e^−^ = 2/2 ratio and another region between pH 8–10, where the slope value decreased to −38 mV/pH, indicating that the process presents a H^+^/e^−^ = 1/2 ratio. The intersection of the data in the graph provides information about the pKa of 5PIBA with an approximate value of 7.8. At pH < pKa, polyindoles undergo a process of 2H^+^/2e^−^ coupling [23,46,57,58,59], and at pH > pKa, the boronic groups are mostly in their anionic form (boronate), contributing to a 1H^+^/2e^−^ process, stabilizing positive charges generated during the p-doping process. Furthermore, it is well known that the acidity of a boronic acid generally increases with a corresponding decrease in its pKa by more than two units. Arylboronic acids have been reported to have a pKa above 8.0 [60,61]. This means that the boron atom would exist in the neutral trigonal form in the free acid form at pH = 7. However, upon ester formation, the boron atom would convert to the tetrahedral anionic form at pH = 7, which is consistent with what has been described for polyindole derivatives functionalized in the same position, where both the pH and the nature of the substituent groups modify the number of protons involved in the redox process. In this sense, the boronic groups of 5PIBA could also establish hydrogen bonding interactions with the indole ring nitrogens (B–OH and N–H), which would explain the transition observed in the pH range, studied previously [23,46,57,58,60,61].

Figure 6c shows the pH vs. ΔEp = (Epc − Epa) graph, where we observe that the peak-to-peak separation of the chemically reversible redox process of 5PIBA varies from 75 to 110 mV between pH 4-7 and that there is an approximate value greater than 150 mV between pH 8-10, this behavior being reported for conducting polymers [62]. Also, when analyzing the variation in the total charge obtained from the CVs as a function of pH (Figure 6d), a significant increase is observed as the pH increases, reaching its maximum value at pH 10. This behavior can be explained by the chemistry of the boronic group, which in its anionic form at pH > pKa favors the accumulation and retention of counterions in the polymer matrix [23]; this effect suggests that the use of 5PIBA in alkaline media could maximize its charge storage capacity.

### 3.7. Evaluation of 5PIBA Interactions with Biomolecules

**Figure 7:**
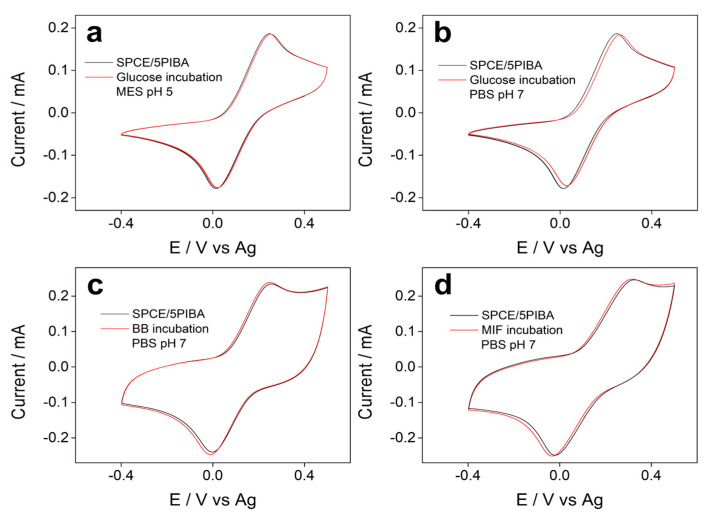
Cyclic voltammograms recorded before (black) and after (red) incubation with (**a**) 1 mg·mL^−1^ glucose (pH 5); (**b**) 1 mg·mL^−1^ glucose (pH 7.0); (**c**) casein 1% (pH 7); and (**d**) 1 μg·mL^−1^ MIF protein (pH 7). All measurements were recorded in 5 mM ferro/ferricyanide at 100 mV·s^−1^.

Figure 7 shows the voltammetric profiles of the electrochemical response of the redox couple [Fe(CN)_6_]^3−^/^4−^ obtained before and after incubation for 1 h with glucose, casein, and MIF (macrophage migration inhibitory factor) protein on SPCE/5PIBA, respectively. In all cases (Figure 7a–d), no variations in peak potentials and current intensities were observed, indicating no interaction between glucose and proteins with the boronic groups under the conditions tested. Glucose was chosen because the most common carbohydrate recognition sensors have been boronic acid-based receptors [60,61]. This is due to the fact that boronic acids react covalently with 1,2- or 1,3-diols to form stable and reversible five- or six-membered cyclic esters. The bonding of diol to boronic acid has been described in two ways in the solution: (1) the bonding of diol to boronic acid with sp2 hybridization and (2) the bonding of diol to boronate with sp3 hybridization. In a basic medium (compared to a pH 7 solution), the tetrahedral boronate is predominant to form the cyclic ester. In our case, the glucose molecule does not have adequate accessibility to the boronic groups, since they are confined in a dense polymeric matrix. The intramolecular stabilization by hydrogen bonds between B–OH groups and the diol is limited to their availability to form the cyclic ester, or the pH-dependent acid–base speciation of the boronic groups, which in polymeric films may differ from their behavior in solution. Another assay carried out was the interaction of 5PIBA with casein (Figure 7c) and MIF (Figure 7d) to test whether these proteins blocked the electroactive surface, thereby suppressing the electrochemical response. The absence of appreciable redox changes after exposure to proteins suggests that 5PIBA could be used in biocorrosion as a protective layer for the biofouling process or as an antimicrobial layer in biological matrices [63]. However, to achieve effective bioconjugation for the development of electrochemical biosensors, it would be necessary to optimize the system and increase surface accessibility, for example through copolymerization with hydrophilic spacers such as polyethylene glycol or polymerization on high-area supports such as nanoparticles or nanotubes. These strategies have proven effective in improving the orientation and availability of boronic groups on the surface, favoring conjugation with diols or glycoproteins [64,65].

The collective outcomes of these investigations substantiate 5PIBA as a versatile, stable, and electrochemically active material, with a property profile that distinguishes it from other conducting polymers previously reported in the literature. Its ability to combine efficient charge storage, high electrocatalytic activity, and the formation of protective coatings opens up a wide range of possibilities in emerging technologies, from energy storage devices to sensing platforms and functional coatings. This potential fully justifies further studies exploring different functionalization strategies, hybridization with nanomaterials, and evaluation in demanding operating environments.

## 4. Conclusions

This work establishes a reproducible electropolymerization route to form adherent, electroactive poly(5-indolylboronic acid) (5PIBA) coatings on carbon and stainless steel substrates. Vibrational and electrochemical evidence indicates polymer formation while retaining the boronic acid groups, yielding pH-tunable interfacial behavior with a pseudocapacitive character. Rather than optimizing device metrics, the study provides a controlled aqueous baseline that clarifies how 5PIBA behaves as a surface coating and enables fair comparisons across substrates and protocols. Within this scope, 5PIBA emerges as a practical platform for electrochemical interfaces, with potential relevance to sensing/biosensing, antifouling, or anticorrosion layers, and charge-storage systems. Looking ahead, this reproducible aqueous route provides a solid springboard for device integration; by optimizing film loading and electrolyte as needed for detailed studies, and coupling 5PIBA with conductive nanostructures, the established interfacial performance can be translated into selective sensing layers and robust charge-storage platforms.

## Figures and Tables

**Table 1:** Contact angle values measured on clean HOPG and 5PIBA-coated substrates.

Surface	Left Angle	Left RMS Error	Right Angle	Right RMS Error	Average Angle
HOPG	80.10°	0.40	78.12°	0.41	79.11°
5PIBA *	36.52°	0.47	41.50°	0.56	38.17°

* Mean of n = 4 drops (independently prepared electrodes).

**Table 2:** Comparison of the specific capacitance of different conductive polymers reported in the literature.

Material	Capacitance (mF∙cm^−2^)	Synthesis	Measurement Solution	Ref.
PEDOT:PSS/ITO	0.83–1.68	Electro/spray coating	ACN/LiClO_4_	[49]
EOG/CCP	0.53–1.5	PECVD ^1^	KOH aqueous	[50]
ProDOT	0.538–1.059	chemical	ACN/LiClO_4_	[51]
p3DMAC-EDOT	4.76	electrochemical	—	[52]
PPy/Cu-MOF	252.1	chemical	KCl aquerous	[53]
PPy/Agar/Polyacrylamide	79.7	chemical	H_2_SO_4_	[53]
PEDOT:PSS	0.23–1.18	chemical	ACN/LiClO_4_	[53]
5PIBA ^2^	0.23	electrochemical	MES buffer	—

^1^ Microwave plasma enhanced chemical vapor deposition. ^2^ this work.

**Table 3:** Parameters obtained from the fitting of the electrochemical impedance spectra in MESbuffer solution pH 5 to OCP and in the presence of Fe(CN)_6_]^3−^/^4−^.

Equivalent Circuit Parameters	SPCE	SPCE/5PIBA	SPCE (10mM FF) *	SPCE/5PIBA (10mM FF) *
Rs (Ω)	3397	2480	150.8	350.6
CPE1–T (F)	1.05 × 10^−6^	1.64 × 10^−4^	1.21 × 10^−6^	1.39 × 10^−3^
CPE1–P	0.94	0.91	0.97	0.76
R1 (Ω)	6.12 × 10^7^	1.58 × 10^6^	383.1	2.88 × 10^−5^
W–R (Ω)	—	—	5504	688,4
W–T (s)	—	—	201.6	2.14
W–P	—	—	0.454	0.231
χ^2^	0.0234	0.0924	0.00088	0.00076

* Fe(CN)_6_]^3−^/^4−^ (FF).

## Data Availability

The original contributions presented in this study are included in the article. Further inquiries can be directed to the corresponding author.

## Abbreviations

The following abbreviations are used in this manuscript:

5PIBA	Poly-5-Indolylboronic Acid
ACN	Acetonitrile
APBA	p-Aminophenylboronic Acid
ATR	Attenuated Total Reflectance
BB	Borate Buffer
BFEE	Boron Trifluoride Diethyl Etherate
CPE	Constant Phase Element
CV	Cyclic Voltammetry
ΔE	Peak Potential Separation
Ea	Anodic Peak Potential
Ec	Cathodic Peak Potential
EIS	Electrochemical Impedance Spectroscopy
FESEM	Field Emission Scanning Electron Microscopy
GCE	Glassy Carbon Electrode
HOPG	Highly Oriented Pyrolytic Graphite
LOD	Limit of Detection
MES	2-Morpholinoethanesulfonic Acid
MIP	Molecularly Imprinted Polymer
OCP	Open Circuit Potential
pKa	Acid Dissociation Constant
Rct	Charge Transfer Resistance
Rs	Solution Resistance
SPCE	Screen-Printed Carbon Electrode
SS	Stainless Steel
χ2	Chi-square
